# Upcycling Coffee Waste into Sustainable Nano Zerovalent Iron for Environmental Contaminant Remediation: Characterization, Applicability and Cytotoxicity

**DOI:** 10.3390/nano15231788

**Published:** 2025-11-27

**Authors:** Filipe Fernandes, Maria Freitas, Cláudia Pinho, Ana Isabel Oliveira, Cristina Delerue-Matos, Clara Grosso

**Affiliations:** 1 REQUIMTE/LAQV, ISEP, Polytechnic of Porto, Rua Doutor António Bernardino de Almeida, 431, 4249-015 Porto, Portugal; fjrfs@isep.ipp.pt (F.F.); mccfs@isep.ipp.pt (M.F.); cmm@isep.ipp.pt (C.D.-M.); 2 Faculty of Sciences, University of Porto, Rua do Campo Alegre, s/n, 4169-007 Porto, Portugal; 3 REQUIMTE/LAQV, School of Health, Rua Doutor António Bernardino de Almeida, 400, 4200-072 Porto, Portugal; clp@estsp.ipp.pt (C.P.); aio@ess.ipp.pt (A.I.O.)

**Keywords:** spent coffee grounds, nano zerovalent iron, low-cost approach, green synthesis, remediation

## Abstract

The agrifood sector produces considerable waste, offering opportunities for sustainable innovation. In the coffee industry, spent coffee grounds (SCG) can be valorized to generate eco-friendly nanomaterials such as nano zerovalent iron (nZVI), widely applied in soil and water remediation. In this study, green nZVIs were synthesized using SCG hydromethanolic extracts and FeCl_3_, subsequently characterized, and assessed for cytotoxicity. High-performance liquid chromatography with diode-array detection (HPLC-DAD) was employed to identify hydroxycinnamic acids, caffeine, and trigonelline in the SCG extracts. Preliminary remediation assays were conducted with seven contaminants, with venlafaxine selected for detailed pH and kinetic studies. Characterization of nZVIs included SEM and EDS analyses, which revealed spherical nZVI particles (72–83 nm) composed of carbon (47%), oxygen (34%), and iron (16%). Dynamic light scattering (DLS) measurements indicated the presence of smaller particles (15–23 nm). Thermogravimetric analysis (TG) confirmed a residual mass of about 20% at 1400 °C. Fourier-transform infrared spectroscopy (FTIR) and X-ray photoelectron spectroscopy (XPS) confirmed phenolic compound incorporation, while X-ray diffraction (XRD) revealed an amorphous structure. The particles exhibited magnetic behavior and showed no cytotoxicity toward MRC-5 and U87 cell lines. Among the tested contaminants, venlafaxine displayed the highest removal efficiency in remediation tests. Compared with chemically synthesized nZVI, the green version exhibited enhanced stability, attributed to the presence of surface-bounded organic matter. Overall, this sustainable and cost-effective approach to produce nZVI from SCG provides an innovative method for waste valorization and environmental remediation.

## 1. Introduction

The agrifood industry generates tremendous amounts of waste, with the coffee sector being a major contributor, with an estimated production of 176.2 million bags in 2024/2025 [1,2]. The coffee industry produces substantial waste, such as spent coffee grounds (SCGs), silver skin, pulp and husk [3]. From harvest to coffee pots, up to 99.8% of all the biomass ends up as waste [4]. Of this, the largest part is SCGs, with estimates between 45 and 90%, which represents around 6 million tons of SCGs produced annually [5,6]. SCGs are produced in domestic settings, commercial establishments, and industrial facilities. Up to 50% of SCGs are produced in small scales. Nonetheless, large-scale coffee producers generate very large amounts of SCGs. A single Nestlé facility in Spain generates 45,000 metric tons of SCGs per year [7]. SCGs are the leftover solid materials that remain after coffee has been brewed. These are made up of roasted and ground coffee beans that, after the brewing process, have been depleted of many of their water-soluble components but still retain a variety of aromatic substances, such as caffeine, tannins, other polyphenols, polysaccharides, melanoidins, and dietary fibers [3]. Their elimination is considered an environmental problem since these compounds can be harmful to soil ecosystems [3,5]. Directly disposing of SCGs in municipal landfills can be problematic, as these can be transformed into toxic substances. SCGs contain oils and additional compounds that can transform the soil into a more acidic form, and their decomposition in landfills also causes larger emissions of greenhouse gases [3,6]. Given these environmental concerns, it is important to investigate alternative methods for recycling and repurposing SCGs [1,2].

Nano zerovalent iron (nZVI) particles have been broadly studied due to their potential in groundwater and soil remediation. These nanoparticles (NPs) can be synthesized by the bottom-up, chemical method. To avoid the use of costly and hazardous reagents, researchers have increasingly focused on green synthesis methods, which employ plant extracts or other natural reducing agents instead of harsh chemicals. Machado et al. [8] produced nZVI from 26 different tree leaf extracts and observed differences in size, agglomeration and settling, and chromium (VI) conversion rates. Green synthesis is an environmentally friendlier and cheaper approach, often resulting in more stable nZVI [9,10]. Martins et al. [11] performed a life cycle assessment to nZVI synthesis and reported that green synthesis presents an environmental impact between 38% and 50% lower, when compared to traditional synthesis, and roughly eight times lower in cost. nZVI have shown promising results in remediating various contaminants, including heavy metals (e.g., cadmium [12], lead [13], arsenic [14]), chlorinated organic compounds [15], nitrates [16], dyes [17] and other organic pollutants [18].

One of the major obstacles in the utilization of nZVI has been its high reactivity and high surface free energy, which can lead to rapid oxidation and passivation when exposed to air or water [19]. Furthermore, nZVI tends to aggregate due to magnetic and van der Waals forces, lowering their surface area and mobility [19]. Another problem is its limited mobility in porous media, which can affect the nZVI’s capability to efficiently remediate contaminated areas [10]. Distinct modifications have been investigated, such as doping with various metals, emulsification, surface coating, and using other materials as supports [20]. Green synthesis of nZVI has great potential in this regard, as it reduces costs and use of toxic chemicals and can additionally enhance stability and improve biocompatibility [10].

Another key aspect of nZVI application is its biocompatibility and potential for ecotoxicity. While numerous studies have explored the effects of nZVI on soil microorganisms [21], soil organisms [22], plants [23], and aquatic life [24], research on the impact of nZVI on human cells remains limited.

In the current study, hydromethanolic extracts from SCGs were produced, which were then used to prepare green nZVI. Conversely to studies by Goren et al. [25] and Park et al. [2], this is the first synthesis of SCG nZVIs without the use of sodium borohydride (NaBH_4_), showing that a completely green synthesis can be achieved with only coffee wastes, reducing the need for toxic solvents. The nZVI was then characterized and compared to nZVI synthesized via the traditional chemical method (C-nZVI), where iron was mixed with NaBH_4_. Furthermore, the cell proliferation of lung cell line MRC-5 and primary glioblastoma U87 cell line in contact with SCG extracts or nZVIs was evaluated. Finally, the green nZVIs were evaluated for their ability to remove several contaminants from water. This paper is aimed at highlighting potential uses of SCGs, the production of environmentally friendlier, more stable, green nZVI, and provides further information on biocompatibility and remediation applications of nZVI.

## 2. Materials and Methods

### 2.1. Materials

Methanol (MeOH) and NaBH_4_ were purchased from Riedel-de Haën (Seelze, Germany). Iron (III) chloride hexahydrate (FeCl_3_) was obtained from Sigma-Aldrich (Steinheim, Germany). Potassium bromide was purchased from Panreac (Barcelona, Spain). Ultrapure water (with resistivities of 18.2 MΩ/cm) was acquired using a Milli-Q water purification system from Millipore (Molsheim, France).

Coffee (*Coffea arabica* L. and *Coffea robusta* L. blend) spent coffee grounds (SCG) were graciously donated by MoCoffee Europe (Azambuja, Portugal). Samples were dried in a dehydrator under 41 °C until less than 5.56 ± 0.53% moisture. Samples were grinded and stored in the dark until further use.

Reagents for total phenolic content (TPC) and antioxidant activity (AA) were acquired from Sigma-Aldrich, St. Louis, MO, USA.

High-performance liquid chromatography (HPLC) standards were purchased from different companies: 4-*O*-caffeoylquinic, 3,5-di- and 4,5-di-*O*-caffeoylquinic (Sigma-Aldrich, St. Louis, MO, USA and Steinheim, Germany), 5-*O*-caffeoylquinic (Alfa Aesar, Karlsruhe, Germany), trigonelline and 3-*O*-caffeoylquinic acid (Extrasynthese, Genay, France), caffeine (Riedel-de Haën, Karisruhe, Germany), and 4- and 5-*O*-feruloylquinic acids (Biopurify, Chengdu, China). Methanol Chromasolv for HPLC was purchased from Riedel-de Haën (Seelze, Germany) and formic acid from Carlo Erba (Val de Reuil, France).

Dimethyl sulfoxide (DMSO) was obtained from Fisher Chemical (Geel, Belgium). Eagle’s Minimum Essential Medium (MEM) was purchased from Sigma-Aldrich (St. Louis, MO, USA). Ethanol was obtained from Fisher Chemical (Waltham, MA, USA). Trypsin and phosphate-buffered saline (PBS) were purchased from Corning (Glendale, CA, USA). Fetal bovine serum (FBS) was purchased from the company Biochrom KG (Darmstadt, Germany). Antibiotic and antimycotic solution (1%) were purchased from Thermo Scientific (Karlsruhe, Germany). Also, 3-(4,5-dimethyl-2-thiazolyl)-2,5-diphenyl-2H-tetrazolium bromide (MTT) was purchased from the company Acros Organics (Beel, Belgium). Other chemicals used were of analytical grade.

Contaminants (venlafaxine hydrochloride, diclofenac sodium solution, sulfamethoxazole, carbamazepine, sulfadiazine and Direct red 80) were purchased from Sigma-Aldrich (St. Louis, MO, USA and Steinheim, Germany) and Reactive blue 5 from BIOSYNTH (Staad, Switzerland).

### 2.2. Preparation of SCG Extracts

Extractions were performed at two different conditions, based on a previous work [26]: (i) SCG40 °C—biomass/solvent ratio of 1 g:50 mL, temperature of 40 °C, extraction duration of 1 h and solvent mixture of H_2_O:MeOH (50/50, *v*/*v*); and (ii) SCG60 °C—ratio of 1 g:100 mL solvent, temperature of 60 °C, for 1 h using H_2_O:MeOH (50/50, *v*/*v*) mixture. After extraction, extracts were filtered with filter paper (FILTER-LAB^®^, Barcelona, Spain), and the solvent mixture was evaporated using a rotary evaporator. The sample was afterwards redissolved in H_2_O:MeOH (50/50, *v*/*v*) to a concentration of 10 mg/mL. Extraction yields were 26.11 ± 1.81% and 29.01 ± 2.38%, for SCG40 °C and SCG60 °C, respectively.

### 2.3. Phenolic Content and Antioxidant Activity

The TPC was assessed, and three AA assays were performed. Folin–Ciocalteu method was chosen to assess TPC, and the AA was measured by 1,1-diphenyl-2-picrylhydrazyl (DPPH^•^) and 2,2′-azino-bis(3-ethylbenzothiazoline-6-sulfonic acid) (ABTS^•+^) radicals, and ferric reducing antioxidant power assay (FRAP), according to Macedo et al. [27], with slight modifications [26].

For all samples, blanks were made using only the solvent, along with negative controls, with all the reagents and solvents used, but without the sample or the standard solution, which were replaced by H_2_O. All assays were performed in triplicate and measured using a plate reader (Synergy HT, Biotek Instruments, Winooski, VT, USA).

### 2.4. High-Performance Liquid Chromatography with Diode-Array Detection (HPLC-DAD) Analysis

A 20 µL aliquot of each extract was analyzed in triplicate on an analytical HPLC unit (Shimadzu Corporation, Kyoto, Japan) previously described [28]. The separation of compounds was performed using a Waters (Wexford, Ireland) C18 Spherisorb ODS2 column (25.0 × 0.46 cm; 5 μm particle size). The mobile phase was composed of 5% formic acid (A) and methanol (B), starting with 5% B. A gradient program was applied as described by Delerue et al. [28], with a solvent flow rate of 920 µL/min. Spectral data were collected for all peaks within the 200–500 nm range, while chromatograms were recorded at 260, 272, and 320 nm. Data processing was carried out using LabSolutions software version 5.82 (Shimadzu Corporation, Kyoto, Japan). Compound identification was achieved by comparing retention times and UV–vis spectra with standards injected under identical conditions. Additionally, spiked samples with available standards were prepared to confirm the identification. External calibration curves were calculated from the areas obtained for each standard at six different concentrations (*n* = 3, each concentration) (Table S1). The identified compounds were quantified with their corresponding standards except for 3,4-di-*O*-caffeoylquinic acid which was quantified as 3,5-di-*O*-caffeoylquinic acid.

### 2.5. nZVI Synthesis

Green nZVI were synthesized by mixing 100 µL FeCl_3_ 0.1 M with 1 mL of extract with a concentration of 10 mg/mL (SCG40 °C or SCG60 °C), under stirring (100 rpm, 15 min), at room temperature, to assure the formation of the NPs. A preliminary study was performed to assess the required volume of FeCl_3_ needed.

An optimized volume of FeCl_3_ (100 µL) was obtained by the highest absorbance measurement at 750 nm and was used for the full interaction with the phenolic compounds existing in the samples. Solvents were evaporated in a dehydrator at 41 °C and the dried NPs were kept in the dark for further use. C-nZVI produced by the chemical method were synthesized by adding 0.02 M NaBH_4_ dropwise to 0.05 M FeCl_3_ at a 1:1 *v*/*v* ratio, under stirring (100 rpm, 30 min), and the next steps were performed in the same manner as for the green nZVI.

### 2.6. nZVI Characterization

(1) Fourier-transform infrared spectroscopy (FTIR): Thermo Scientific Nicolet 6700 FT-IR spectrometer (Thermo Fisher Scientific, Waltham, MA, USA) was used to obtain the IR spectra of the samples. The sample and KBr were combined at a ratio of 1:100 and compressed under high pressure. They were immediately analyzed in the range of 450–4000 cm^−1^.

(2) UV-vis spectroscopy: The UV-Vis spectrum of the nZVI was detected utilizing a Shimadzu UV-1900i spectrophotometer (Shimadzu Corporation, Kyoto, Japan), at wavelengths between 190 and 400 nm.

(3) Scanning Electron microscopy (SEM) and elemental analysis: The stub was coated with a silicon base to deposit the sample and perform the SEM, elemental and mapping analysis prior to the NPs deposition. To determine the nZVI particles’ morphology, size and composition, a SEM Quanta 400 FEG was used (FEI, Hillsboro, OR, USA) under the following conditions: secondary electron and a back-scattered electron detectors, accelerating voltage (15 KV), magnification (10× up to 400.000×), and low vacuum. For elemental analysis, EDS spectra were obtained using the same electron microscope with a detector type SUTW SAPHIRE analysis system of resolution 132.19 (coupled with an EDAX Genesis X4M Energy-dispersive Spectroscopy, FEI, Hillsboro, OR, USA). The microscopy analyses were performed at Centro de Materiais da Universidade do Porto (CEMUP), Portugal.

(4) Dynamic Light Scattering analysis (DLS): The nZVI size, its distribution (polydispersity index—PDI) and zeta potential parameters were analyzed by DLS using a NanoZS (Malvern Instruments, Ltd., Malvern, UK). nZVIs were dispersed in ultrapure H_2_O (1:5 mg nZVI, mL solvent), and ultrasound (10 min) was used to disperse the nZVI unanimously. Tween^®^20 (0.2%) was used as a surfactant to prevent agglomeration and settlement. Malvern DTS1061 cuvettes were used, and 3 cycles of 10 runs each were used for size measurement. For zeta potential, 5 cycles of 10 runs each were used.

(5) X-ray diffraction (XRD) analyses: Samples were ground and mounted on standard powder sample holders. XRD analyses were carried out at room temperature using a PANalytical X’Pert Pro diffractometer (Malvern Instruments, Ltd., Malvern, UK), equipped with an X’Celerator detector and a secondary monochromator. The energy used to produce the X-radiation was 40 kV and 30 mA. Data acquisition was performed in Bragg–Brentano geometry between 10° < 2θ < 90°, with CuKα radiation (λ1 = 1.54060 Å and λ2 = 1.54443 Å), 0.017°/step, 100 s/step). The analysis was performed at Unidade de Microscopia Eletrónica (UME), at Universidade de Trás-os-Montes e Alto Douro, Portugal.

(6) X-ray photoelectron spectroscopy (XPS) analysis: XPS was used to reveal the elemental compositions and oxidation state of the surface of the synthesized nZVI. This was performed on Kratos Axis Ultra HAS (Manchester, UK), equipped with a monochromator and an X-ray power source of 15 kV. The analyses were performed at Centro de Materiais da Universidade do Porto (CEMUP), Portugal.

(7) Thermogravimetric analysis (TG): TG analysis was conducted using a Netzsch STA 449 F3 Jupiter (Netzsch, Selb, Germany). Samples were deposited in aluminum oxide crucibles and elevated from room temperature to 1400 °C at a rate of 20 °C min^−1^ under air atmosphere.

(8) Magnetic properties: The magnetism of the synthesized nZVIs was evaluated using a superconducting quantum interference device magnetometer (SQUID), at the Physics and Astronomy Department, Faculty of Sciences of Porto University (IFIMUP). Magnetization as a function of the applied magnetic field was used to study the magnetic properties.

(9) Stability: To assess nZVI stability in suspension, nZVI without the addition of Tween^®^ 20, with the addition of Tween^®^ 20 before synthesis (B4), and with the addition of Tween^®^ 20 after synthesis (Af) were kept at 4 °C and at 20 °C. Stability was analyzed by UV-Vis and FTIR analysis. Analyses were made right after synthesis, and after 1 h, 1 day, 1 week, two weeks, and one month. These results are presented in Figures S1 and S2.

### 2.7. In Vitro Cytotoxic Activity

Lung cell line MRC-5 (normal) and immortalized human primary glioblastoma cell line (U87 cell line) were obtained from the American Type Culture Collection (ATCC) (Barcelona, Spain). Cells were maintained in culture with Eagle’s Minimum Essential Medium (EMEM) (MRC-5) or RPMI (U87) supplemented with 10% FBS and 1% antibiotic-antimycotic solution and incubated at 37 °C and 5% CO_2_. Subcultures were performed when cell lines reached 80% confluence.

Cell viability was determined by MTT colorimetric assay [29,30]. Cells were seeded in 96-well plates at a density of 2.0 × 10^5^ cells/mL. Following 48 h incubation and attachment, medium was taken out, and cells were treated with SCG40 °C extract and SCG40 °C-nZVI at different concentrations (0.2–500 µg/mL), for 24 h. MTT assay was performed by adding 10 µL MTT (5 mg/mL) to each well and left incubating for 1 h, at 37 °C in the CO_2_ incubator. At the end of the incubation time, and after removing the supernatant, 100 μL of DMSO:absolute ethanol (1:1) was added to each well to dissolve the formazan crystals. Absorbance (OD) was measured at 570 nm using a microplate reader (Thermoscientific^®^ MULTISKA FC). The results were expressed as the percentage of control, by the formula (As − Ab)/(Ac − Ab) × 100, where As is the absorbance of the sample, Ab is the absorbance of the blank, and Ac is the absorbance of the control.

### 2.8. Environmental Remediation Assays

To assess the remediation potential of the synthesized nZVI, SCG40 °C-nZVIs were applied to a series of contaminants, venlafaxine (Vlx), diclofenac (Dfc), sulfamethoxazole (Smx), carbamazepine (Cmz), sulfadiazine (Szn), Reactive blue 5 (RB5), and Direct red 80 (DR80). Calibration curves were constructed for each contaminant, with values between 1.0 × 10^−6^ and 25 mg/L. For the remediation assay, 4 mL of a contaminant solution (25 mg/L) was added to 5 mg of SCG40 °C-nZVI. The mixtures were continuously stirred, and the absorbance was measured after 30 min and 60 min at 224 nm (Vlx), 264 nm (Szn), 267 nm (Smx), 276 nm (Dfc), 285 nm (Cmz), 528 nm (DR80) and 603 nm (RB5). All assays were performed in triplicate and measured using a microplate reader (Synergy HT, Biotek Instruments, Winooski, VT, USA).

For Vlx, additional experimental assays were carried out: (a) by varying the pH of contaminant solution (pH from 2 to 12); and (b) by varying the time of contact (10, 20, 25, 30, and 60 min).

For the kinetic studies, the initial concentration of Vlx (*C*_0_) was 25 mg/L, the volume of solution (*V*) was 0.025 L and the mass of nZVIs (*m*) was 0.025 g.

The amount of adsorbed Vlx in each time (*Q_t_*, mg/g) was calculated using Equation (1):

(1) $$Q_{t}=\frac{C_{0}-C_{t}}{m}\times V$$

Two kinetic adsorption models were fit to the experimental results, namely pseudo-first order (PFO, Equation (2)) and pseudo-second order (PSO, Equation (3)) models [31,32]. The corresponding equations are below:

(2) $$Q_{t}=Q_{e}\left(1-e^{-k1\times t}\right)$$

(3) $$Q_{t}=\frac{k2\times {Qe}^{2}\times t}{1+k2\times Q_{e}\times t}$$

where *Q_t_* is the adsorbed amount of the adsorbate at time *t* (mg/g), *Q_e_* is the adsorbed amount of adsorbate in equilibrium (mg/g), *k*_1_ and *k*_2_ are, respectively, pseudo-first-order rate constant (min^−1^) and pseudo-second order rate constant (g.mg^−1^.min^−1^), and *t* is adsorption time (min).

The model fitting was performed using Excel Solver (version 2507 Build 16. 0. 19029. 20136) by assigning values to *Q_e_*, *k*_1_, and *k*_2_, with the best fit determined through the minimization of the sum of squared errors (SSE).

### 2.9. Statistical Analysis

The results are presented as the mean ± standard deviation based on a minimum of three replicates. A *t*-test was conducted to measure TPC and AA between both extracts using GraphPad Prism (version 8.0.1). For comparison between green nZVIs and ZVIs obtained through the chemical method, an ANOVA with Tukey post hoc test was carried out. In both cases, the statistical significance was set as *p* < 0.05.

## 3. Results and Discussion

### 3.1. HPLC-DAD Analysis of the SCG Extracts

**Figure 1:**
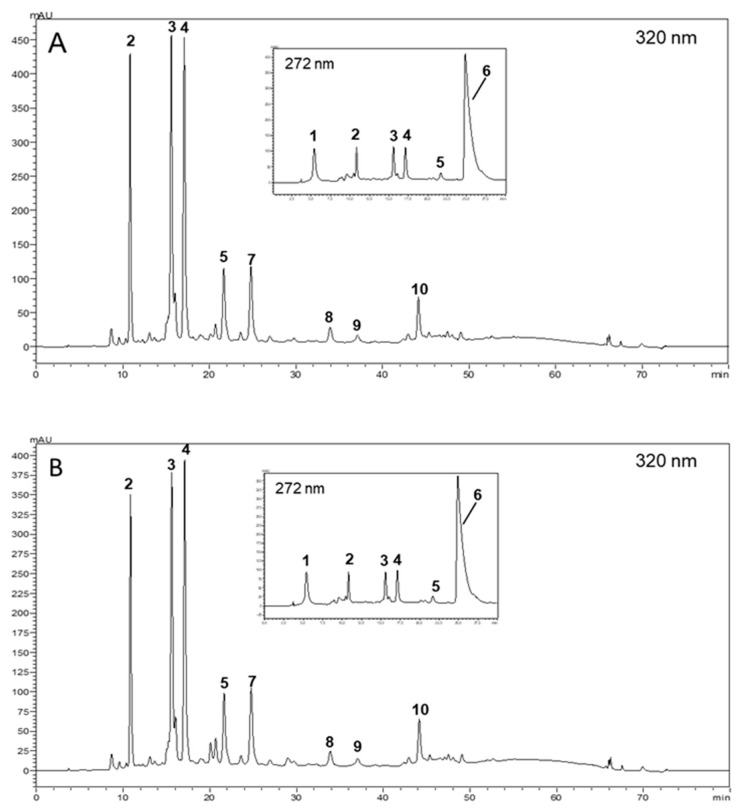
HPLC-DAD chromatograms of SCG40 °C (**A**) and SCG60 °C (**B**) obtained at 320 nm. A detail of the first 30 min elution recorded at 272 nm is also provided. Peak identity as in Table S1.

Ten compounds were identified and quantified in SCG40 °C and SCG60 °C extracts: three caffeoylquinic acids (CQA)—3-*O*-CQA (2), 4-*O*-CQA (3) and 5-*O*-CQA (4); two feruloylquinic acids (FQA)—4-*O*-FQA (5) and 5-*O*-FQA (7); three dicaffeoylquinic acids (di-CQA)—3,4-di-*O*-CQA (8), 3,5-di-*O*-CQA (9) and 4,5-di-*O*-CQA (10); trigonelline (1) and caffeine (6) (Figure 1). These compounds were already identified by other authors in SCG from both coffee species (*Coffea arabica* L. and *Coffea canephora* var. *robusta* L.) [33,34,35].

Caffeoylquinic and feryloylquinic acids dominated both extracts. Caffeine was also abundant, with concentrations between 41.68 and 47.50 mg/g extract dw (Table 1). All the other identified compounds were in lower amounts. SCG40 °C was the richest extract in all compounds (*p* < 0.05), except for 3,5-di-*O*-CQA, found in similar concentrations in both extracts (Table 1). The reported amounts of CQAs, FQAs, di-CQAs, and caffeine in SCG extracts vary widely across published studies. For example, Angeloni et al. [33] investigated the composition of four different extracts from 100% *C. arabica* SCG from Ethiopia. Among the compounds identified—hydroxybenzoic and hydroxycinnamic acids, flavonoids, xanthones, stilbenes, secoiridoid glycosides, and caffeine—caffeine was found in significant amounts, followed by 5-*O*-CQA, 3-*O*-CQA, and 3,5-di-*O*-CQA. While the caffeine content aligns with the current study, 5-*O*-CQA and 3-*O*-CQA levels were lower. Another study by Bravo et al. [34] compared SCG extracts from *C. arabica* and *Coffea canephora* var. *robusta* prepared in water after defatting with petroleum ether using a Soxhlet device. The quantification revealed that SCG extracts from *C. arabica* had higher CQAs and di-CQAs levels than those from *C. canephora* var. *robusta*, although all compounds were present in lower amounts than those reported in Table 1. Similarly, caffeine levels varied from 3.59 to 8.09 mg/g in spent coffee, which were also 5 to 10 times lower.

### 3.2. Evaluation of Phenolic Compounds and Their Antioxidant Properties

The reduction of Fe^3+^ to Fe^0^ enables the synthesis of nZVI, and thus the evaluation of the reducing capacity of the SCG extracts is crucial for producing environmentally friendly green nZVI. The results for TPC, DPPH^•^ and ABTS^•+^ scavenging activities, and FRAP assay are presented in Table 2. SCG40 °C displayed the best results for TPC (*p* = 0.0076) and DPPH^•^ scavenging assay (*p* = 0.0174), while no statistically significant differences were found for FRAP method and ABTS^•+^ scavenging activity. The TPC values are above those usually reported in the literature, with Ballesteros et al. [36] having stated a TPC of 40.36 mg GAE/g SCG, and Solomakou et al. [37] reporting a TPC of 34.43 mg GAE/g SCG. However, Abdeltaif et al. [38] reported a TPC value of 97.87 mg GAE/g SCG. Andrade et al. [39] produced SCG extracts by SFE, UAE and soxhlet with different solvents, and achieved TPC values ranging from 24.1 to 57 in the SFE extractions, Between 221.5 and 587.7 in the UAE extractions, and 119.5 to 182.6 mg chlorogenic acid equivalents/g sample for Soxhlet extractions. The difference in the UAE and Soxhlet extractions can be explained by the choice of solvent, with dichloromethane presenting the lowest TPC and ethanol the highest in UAE, and ethanol yielding the lowest values and ethyl acetate the highest in Soxhlet. In the SFE extractions, different pressures and the addition of ethanol were assessed, with 4% ethanol, at 200 bar and 323.15 K resulting in the highest TPC, while a pressure of 200 bar and 323.15 K resulting in the lowest TPC. Ballesteros et al. [36] reported an AA of 28.15 mg TE/g SCG, 31.54 mg TE/g SCG, and 68.58 mg Fe(II)/g SCG for DPPH^•^, ABTS^•+^, and FRAP methods, respectively, for extracts produced by autohydrolysis, with SCG40 °C and SCG60 °C behaving as better antioxidant samples. Andrade et al. [39] reported ABTS^•+^ values between 48.7 and 275.1 μMTEAC/g. The results imply that the choice and volume of solvent, along with the extraction method display a significant influence in the number of phenolic compounds extracted and AA.

### 3.3. FTIR Analysis

**Figure 2:**
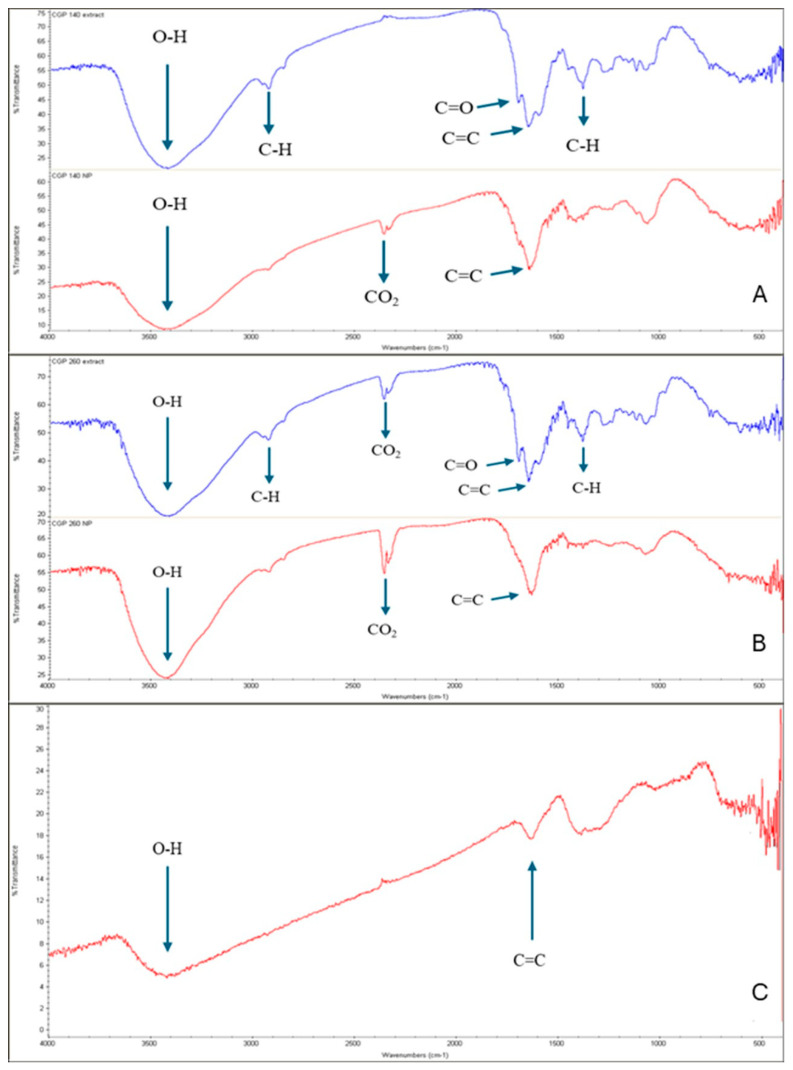
FTIR spectra of extracts (red) and nZVIs (blue). (**A**) SCG40 °C extract and nZVI; (**B**) SCG60 °C extract and nZVI; (**C**) C-nZVI.

FTIR analysis was performed for both extracts and nZVIs. The data is presented in Figure 2A–C. The SCG extracts (Figure 2A,B, in red) showed a peak at 3400 cm^−1^, related to OH groups of polyphenols [40,41]. The reduced intensity might be explained by the oxidation of the OH groups and formation of an iron–phenol complex [42]. The peaks at 2930 cm^−1^ indicate the CH and CH_2_ vibrations of aliphatic hydrocarbons, and the peaks displayed at 1700 cm^−1^ are assigned to C=O stretching vibrations of carbonyl groups [43]. The peaks at 1650 cm^−1^ are attributed to C=C stretching vibrations [44]. The bands in the 1450–1000 cm^−1^ area, such as those around 1385, 1285, and 1122 cm^−1^, can be attributed to chlorogenic acids, as these display strong absorption bands in this area and are responsible for the reduction in the Fe^3+^ to Fe^0^ [45].

Mohamed et al. [45] observed that the changes in the spectra of the nZVI indicate the interaction of Fe with the phenolic compounds of the extract. The FTIR spectra for SCG-nZVI (Figure 2A,B, in blue) display a broadened peak at 3400 cm^−1^, assigned to the OH stretching vibrations, which indicate the presence of phenolics [41]. The peak at 2930 cm^−1^, attributed to CH and CH_2_ vibration of aliphatic hydrocarbons is smaller than in the extract, which indicates the interaction of the nZVI with the extracts [46]. The peak that was present at 1700 cm^−1^ in the extracts is no longer present in the nZVI, which, along with the absence of the peak at 1385 cm^−1^, is attributed to the interaction of Fe with the extract components [45]. There is a peak at 1650 cm^−1^, attributed to the C=C stretching vibrations [44]. The broadened peak at 1100 cm^−1^ is attributed to C–O, C–O–H, and symmetric and asymmetric C–O–C groups of the nZVI [46].

### 3.4. UV-Vis Spectroscopy and Thermogravimetric Analysis

**Figure 3:**
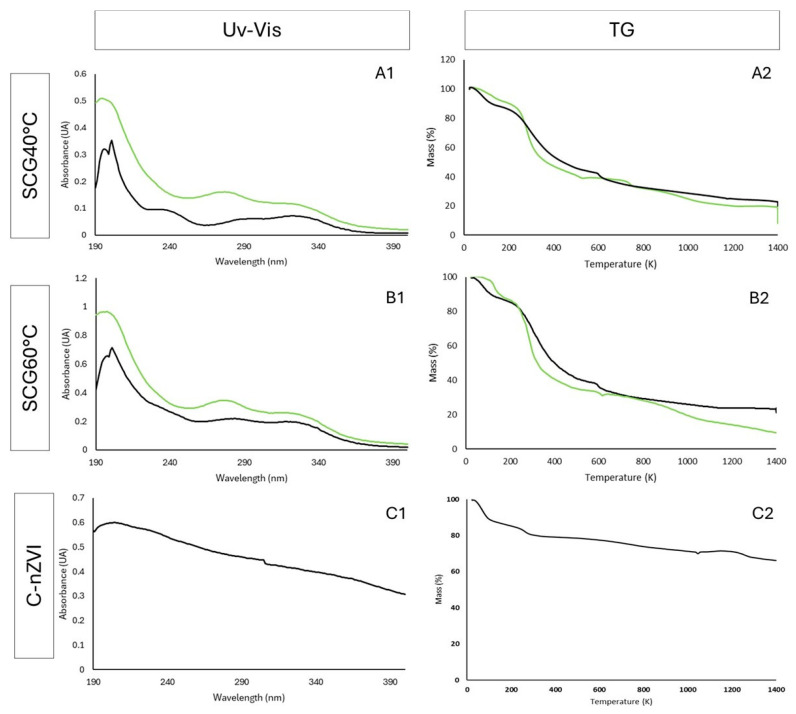
Analysis of SCG40 °C extract and nZVI (**A**), SCG60 °C extract and nZVI (**B**), and C-nZVI (**C**). UV-Vis spectra (**A1**,**B1**,**C1**) and TG measurements (**A2**,**B2**,**C2**). Extracts are presented by the green line and nZVI with a black line.

The spectra obtained by UV-Vis for the SCG extracts, nZVI and C-nZVI are presented in Figure 3(A1–C1). Both SCG-nZVI (Figure 3(A1,B1), black line) displayed their highest peak at 201 nm, which is in line with previous studies which indicated nZVI peaks are usually located between 200 and 400 nm [40,42]. The absorbance peak depicts the polyphenols extracted from the SCG [47]. The extracts and the nZVI absorption peaks displayed some similarities, which confirms the existence of compounds from the extracts on the nZVI. Abdelfatah et al. [43] produced nZVI from *Ricinus communis* L. seeds extract and reported an increase in the absorption spectrum at approximately 300 nm, which was ascribed to the development of nZVI. The C-nZVI (C1) displayed a distinct spectrum, which further exemplifies that in SCG-nZVI organic matter is present.

**Figure 4:**
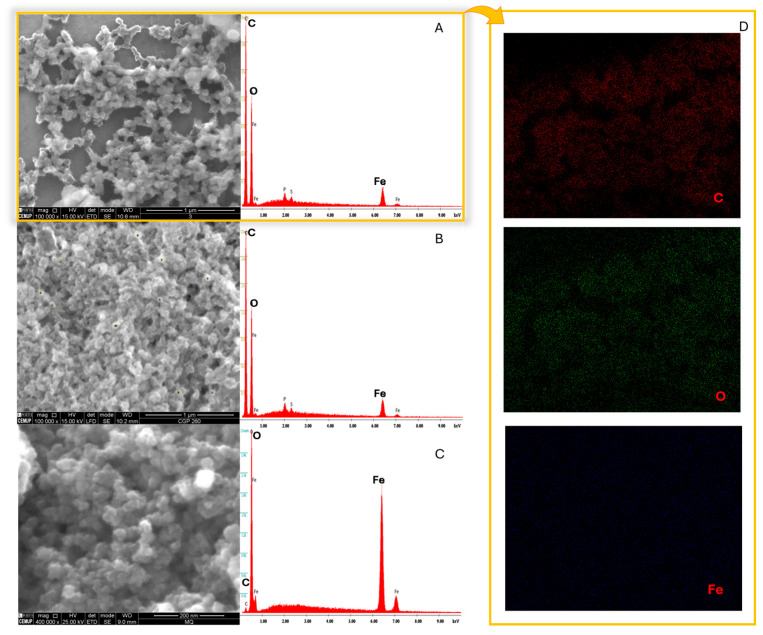
SEM images and EDS spectra of synthesized SCG40 °C-nZVI (**A**), SCG60 °C-nZVI (**B**) and C-nZVI (**C**). Elemental mapping analysis of synthesized SCG40 °C-nZVI (**D**).

Thermal analysis of the extracts and nZVI were conducted (Figure 3(A2–C2)). The first step, until around 200 °C, can be attributed to the dehydration of the materials. Afterwards, there is a steady loss in mass, attributed to a loss of volatilized phenolic compounds across the test. The C-nZVIs (Figure 3(C2)) displayed a much lower loss in mass, since they possess no phenolic compounds on the surface of the nZVI, and the iron is not destroyed during the analysis [48]. The SCG nZVI (Figure 3(A2,B2), black line) stabilizes at roughly 20% of the initial mass, after the breakdown of the outer organic carbon shell, leaving only elemental iron present. This is in line with EDS analysis, which indicates an iron content of approximately 16%. The nZVI displayed higher thermal stability when compared to the SCG extracts, as the extracts lose mass at lower temperatures than the nZVI. The results are presented in Figure 4A–C. Lem et al. [49] produced nZVI from onion peel extract and performed a TG analysis. However, the nZVI were freeze-dried prior to the analysis, therefore there was no initial weight loss due to water molecules evaporating. Nonetheless, the comparison between the onion peel extract and the nZVI was similar to the results attained in the present work, with a steady mass loss, with higher stability in the nZVI, and at 800 °C, 60% of the initial weight remained in the nZVI, compared to 40% in the extract.

### 3.5. SEM/EDS Analysis

Figure 4A–C exhibit the SEM micrographs of the produced nZVI. The analyses were conducted using a stub coated with a silicon base to perform the mapping and elemental analysis to avoid carbon interference of a typical carbon stub. ImageJ (version 1.8.0) images demonstrate that nZVI displayed spherical shape, with average sizes of 83.50 ± 14.69 nm and 72.47 ± 12.56 nm for SCG40 °C-nZVI and SCG60 °C-nZVI (*p* = 0.0044), respectively. Both SCG extracts produced similar NPs (Figure 4A,B), whereas C-nZVI (Figure 4C) were of a regular, round shape, with average sizes of 34.92 ± 7.11 nm, and a lower size dispersity, being statistically different from the green nZVIs (*p* < 0.0001). The EDS spectra revealed similar results for both SCG nZVI (Figure 4A,B), with pronounced peaks related to carbon and oxygen (approximately 47% and 34%, respectively), confirming the presence of organic compounds on the nZVI surface. Iron was the third largest compound present, roughly 16%, with about 1 and 0.6% of phosphorus and sulfur, respectively. The C-nZVI displayed a sharply different EDS spectrum, with 80% iron and 20% oxygen, absence of carbon, and therefore no organic matter present. Desalegn et al. [42] prepared nZVI from mango peel extracts and reported amorphous NPs with 48.5% of iron, along with 34.06% and 14.95% of oxygen and carbon, respectively. This shows a lower amount of organic matter on the surface of the nZVI. Abdelfatah et al. [43] produced nZVI from R. communis seeds extract, and reported nZVI as granular with a spherical shape, average particle sizes of 20 nm, and Fe, C, and O contents of 7.11, 34.36, and 46.16%, respectively. The reported studies show that the extract used has a meaningful influence on the characteristics such as size, shape, and elemental composition of the produced nZVI. A subsequent analysis was performed to corroborate the elemental analysis. The presence of carbon and oxygen are clearly visible in the mapping analysis, which is in accordance with the EDS results.

### 3.6. Dynamic Light Scattering Analysis

DLS is used to provide fast, non-destructive measurements of particle size distributions in the nanometer range. It also presents, however, some challenges, mainly a high sensitivity to large aggregates, which makes it a difficult assay to apply when measuring nZVI [50]. The application of a dispersant could considerably improve DLS analysis. Bhattacharjee [51] found that the choice of solvent significantly influences DLS measurements, as stable and highly monodisperse carboxylated latex beads dispersed in different solvents—40% sucrose, water, methanol, and toluene—exhibited average particle sizes of 15, 87, 122, and 153 nm, respectively.

Water was used as the dispersing medium, and Tween^®^20 served as a surfactant to minimize aggregation by reducing hydrophobic interactions between particles. Details of the results can be found in Table 3. Large aggregates were present in the SCG40 °C-nZVI and SCG60 °C-nZVIs when the analyses were performed without Tween^®^20 (Table 3). Afterwards, Tween^®^20 (0.2%) was added, and the mean particle size measured was 14.64 ± 0.76 nm, with a PDI of 0.24 ± 0.07. When Tween^®^20 was added to the methanolic dispersion, the mean particle size increased to 2112.33 ± 483.02 nm, which correlates with previous studies that reported larger particle sizes when dispersion was made in organic solvents. These results highlight that water is a better dispersant, so further assays were performed using water. SCG-60 °C-nZVIs were analyzed in an aqueous solution containing Tween^®^20 (0.2%) and displayed similar results to SCG-40 °C-nZVI, with a mean particle size of 22.68 ± 6.79 nm and PDI of 0.24 ± 0.08. C-nZVI displayed large particle sizes, even with the addition of Tween^®^20 (868.16 ± 142.12 and 466.86 ± 24.52, respectively), which is not in accordance with SEM observations. This might be due to higher aggregation, which results in large clusters. Furthermore, the scattering power of dispersed particles increases with the 6th power of the particle diameter, and few large particles can mask many small particles [52]. Its model assumes spherical particles, and the existence of other shapes greatly impacts the results. The NP translational diffusion coefficient depends not only on the size, but the kind of ions and their concentration on the surface of the NP. Therefore, DLS measurements may not provide an accurate representation of size in all cases, especially if the surface properties of the particles vary or if the system contains diverse types of ions. Finally, in polydisperse systems, DLS reports an average diameter, which further limits its accuracy for size measurements [53].

Mahmoud et al. [54] synthesized nZVI from green coffee extract. The nZVI were analyzed by DLS and displayed a hydrodynamic size of 1884 nm, with a PDI of 0.563 and zeta potential (ZP) of −30 mV. However, when analyzed by electron microscopy (FESEM, HRTEM and STEM), the size of the nZVI ranged from 1 to 30 nm, with mean size of 4 nm. Ruiz-Torres et al. [55] synthesized nZVI by reducing the FeCl_3_ in MeOH using NaBH_4_ as the reducing agent. Furthermore, ethylene glycol was used as a coating agent to reduce polydispersity. The obtained coated nZVI displayed a particle size of 6.5 nm, and ZP of +6.3 mV, while the uncoated nZVI displayed a particle size of 265.1 nm and ZP of −13.0 mV.

Zeta potential was also measured, and the addition of Tween^®^20 influenced the ZP of nZVI, increasing from −19.57 ± 0.95 mV to approximately −5.99 ± 1.71 mV in the SCG-40 °C-nZVI samples with the surfactant. Somchaidee and Tedsree [55] produced nZVI from guava leaf extract and obtained nZVI with sizes between 1.8 and 3.9 nm, with ZP between −40 and −45 mV, indicating a stable system. Tesnim et al. [40] analyzed nZVI produced from palm petiole extract and obtained a mean particle size of 72 nm, with a ZP of −7.2 mV. Despite that, the PDI obtained was 1, which indicates a very high polydispersity, with large aggregates being present. Abdelfatah et al. [43] analyzed nZVI produced from a *R. communis* extract by DLS, obtaining particle sizes between 40 and 50 nm, compared to size distribution between 10 and 30 nm for TEM micrographs. The synthesized nZVI displayed a ZP of −14.9, compared to −2.43 mV for uncoated nZVI.

### 3.7. X-Ray Diffraction Analysis

**Figure 5:**
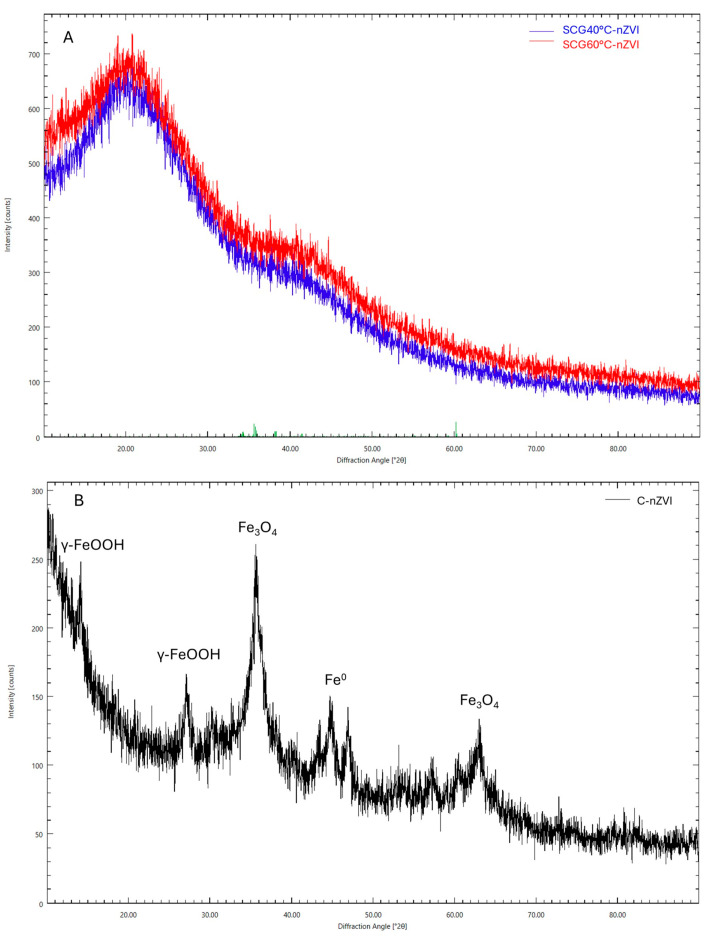
XRD patterns of synthesized SCG40 °C-nZVI and SCG60°-nZVI (**A**), and C-nZVI (**B**).

XRD analysis of the synthesized SCG nZVI was conducted, and the results are presented in Figure 5A. Both types of nZVI display XRD patterns with no obvious crystalline structure. This is due to the phenolic compounds forming complexes with iron, as previously reported [42]. C-nZVI (Figure 5B) displayed a starkly different XRD pattern, as no organic matter is used in its synthesis. The Fe^0^ peak can be seen at 2θ = 45°, as previously reported. The largest peak is found at 2θ = 36°, with some lower peaks at 2θ = 27, 47, and 63°. These are attributed to the formation of iron oxides and hydroxides, mainly magnetite (Fe_3_O_4_) and lepidocrocite (γ-FeOOH) [56].

### 3.8. X-Ray Photoelectron Spectroscopy Analysis

**Figure 6:**
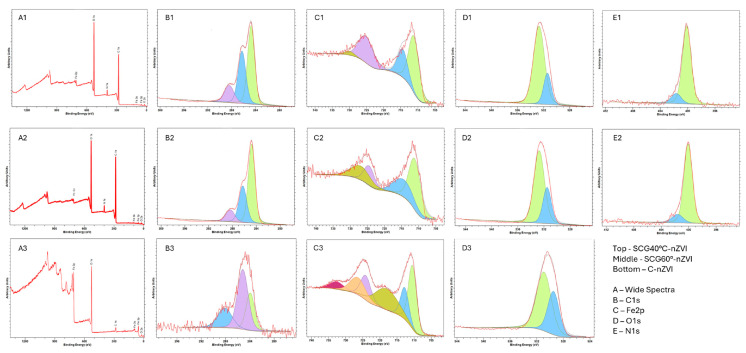
XPS spectra of synthesized SCG40 °C-nZVI (**top**), SCG60°-nZVI (**mid**) and C-nZVI (**bottom**). (**A1**–**A3**) correspond to the wide spectra. (**B1**–**B3**) are the C1s spectra, while (**C1**–**C3**) correspond to the Fe2p spectra. (**D1**–**D3**) correspond to the O1s spectra, while (**E1**,**E2**) are the N1s spectra.

The XPS spectra of the synthesized nZVI are present in Figure 6. The wide spectra of SCG40 °C-nZVI (Figure 5(A1)) and SCG60 °C-nZVI (Figure 5(A2)) are very similar, while the spectra of C-nZVI (Figure 5(A3)) is very different from the former two. The larger peaks in the SCG nZVI correspond to O1s and C1s, due to the presence of the organic matter, while the larger peaks for C-nZVI correspond to O1s and Fe2p. XPS is a surface analysis with a probing depth of 2–5 nm [57,58], which explains the low iron peak in SCG nZVI, as the phenolic compounds are present on the surface of the nZVI, masking the iron core. The C1s (B1–B3) spectra display the sp^2^ C–C, sp^3^ C–C and C = O peaks at 284.8 eV, 286.3 eV, and 288.4 eV, respectively [59]. The sp^2^ content was higher in the SCG nZVI, approximately 50%, while in the C-nZVI it was only 25.9%. On the other hand, the sp^3^ content was over 50% in the C-nZVI, while it was approximately 33% in SCG nZVI. The O1s (Figure 6(D1,D2)) peak of the SCG nZVI was deconvoluted into two peaks, 531.5 and 532.7 eV, which can be attributed to the C-O and C=O groups, respectively. The C-nZVI O1s (Figure 6(D3)) spectra showed two peaks, at 530.9 and 529.5 eV, which can be attributed to the Fe-O bond of iron oxides [59,60]. The N1s peak was not present in the C-nZVI, but was present in the SCG nZVI (Figure 6(E1,E2)), at 400.1 and 401.7 eV, corresponding to N-H and C-N, displaying the presence of compounds from SCG on the surface of the nZVI [61]. The Fe2p spectra were deconvoluted into 4 peaks in the SCG nZVI (Figure 6(C1,C2)). The peaks were seen at 711.2 eV (Fe (II) 2p_3/2_), 714.5 eV (Fe (III) 2p_3/2_), 725.0 eV (Fe (II) 2p_1/2_), and 728.5 eV (Fe (III) 2p_1/2_). The C-nZVI deconvoluted peaks (C3) were present at 710.5 eV (Fe (II) 2p_3/2_), 712.8 eV (Fe (III) 2p_3/2_), 718.0 eV, 724.2 eV (Fe (II) 2p_1/2_), 726.5 eV (Fe (III) 2p_1/2_), and 732.6 eV (Fe (III). The 718.0 eV peak can be attributed to Fe^0^ 2p_1/2_ [13,62]. The absence of the Fe^0^ peak in the SCG nZVI can be explained by the presence of phenolic compounds and the iron oxides, which form a layer surrounding the Fe^0^ core [61].

### 3.9. Magnetic Properties

**Figure 7:**
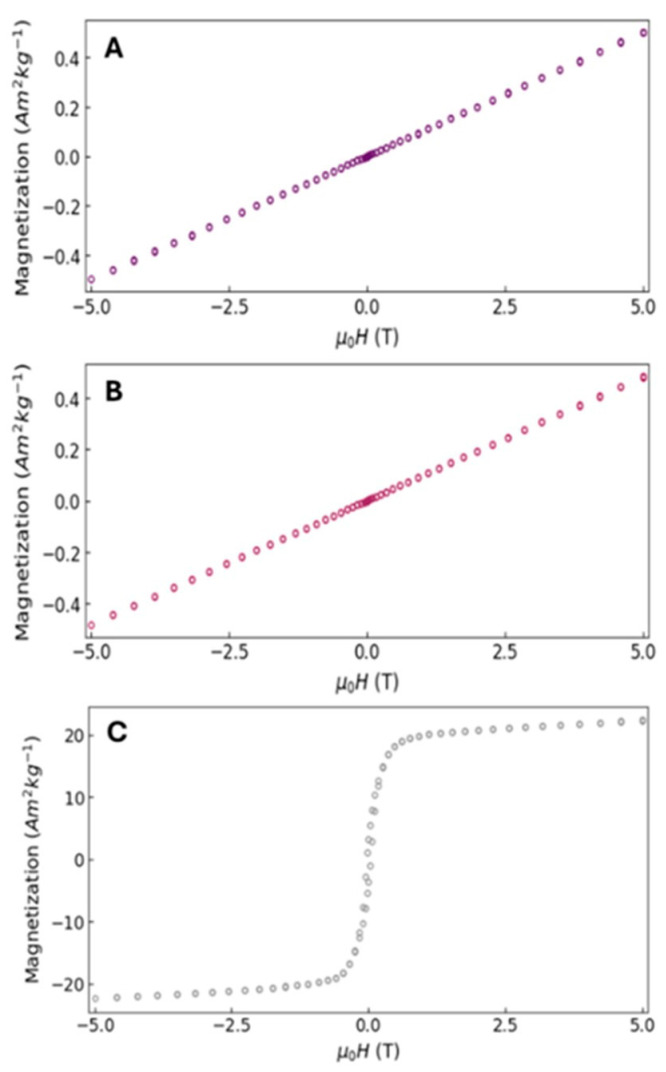
Magnetization (Am^2^Kg^−1^) vs. applied magnetic field (µ_0_H) of the synthesized nZVI obtained by the green method using ground coffee (**A**) SCG40 °C-nZVI, (**B**) SCG60 °C-nZVI, and comparison with the chemical method (**C**).

The magnetic properties of the nZVI were evaluated to investigate the magnetic state of each material at 300 K, and the results are presented in Figure 7. A magnetic field (µ_0_H) was applied, and the magnetization (Am^2^Kg^−1^) was recorded. The magnetic susceptibility (χm) of the synthesized material was comparable between the tested nanoparticles exhibiting a lower magnetic moment (Figure 7A,B), with χm = 1.26 × 10^−7^ m^3^Kg^−1^ for the SCG40 °C-nZVI and χm = 1.21 × 10^−7^ m^3^Kg^−1^ for the SCG60 °C-nZVI. A slight increase in magnetization was observed for SCG40 °C-nZVI, consistent with its slightly larger particle size relative to SCG60 °C-nZVI. These findings indicate that magnetism increases with particle size and surface area. Overall, the data indicate that the green-synthesized nanoparticles are paramagnetic, exhibiting no saturation magnetization, which is expected due to the presence of surface organic matter. This behavior contrasts with chemically synthesized nZVIs (Figure 7C), which typically display superparamagnetic characteristics with saturation magnetization values of approximately 20 Am^2^Kg^−1^).

Despite the relevance of characterizing the magnetic properties of nZVIs produced from SCGs, only a limited number of studies have explored this technique. Mahmoud et al. [54] evaluated the magnetization of green coffee-based NPs and compared it with clove-based NPs, reporting a higher magnetization for the green coffee sample, explained by the larger particle size and surface area; however, no saturation magnetization was achieved. In contrast, Ashaf et al. [63] described a superparamagnetic response in green-synthesized iron-oxide nanoparticles stabilized with black coffee extract, reporting high saturation magnetization (21.72 emu g^−1^).

### 3.10. Stability

UV-Vis and FTIR analyses were conducted to assess the stability of nZVI in solution. nZVIs were kept at room temperature and at 4 °C for up to one month. The influence of Tween^®^20 was also analyzed. The results are presented in Figures S1 and S2. SCG40 °C-nZVI did not show significant differences in the UV-Vis spectra, over the course of one month. The same can be said for the FTIR analysis, with the same peaks being present, which implies that even in suspension, the nZVIs are stable, and no significant alterations in their structure occur. Despite this, significant sedimentation occurs, as can be seen in Figure S3. The picture shows nZVIs after synthesis (Figure S3A), after 1 day (Figure S3B), and after 1 week (Figure S3C). Sedimentation is clearly visible after 1 h.

### 3.11. Cell Viability

**Figure 8:**
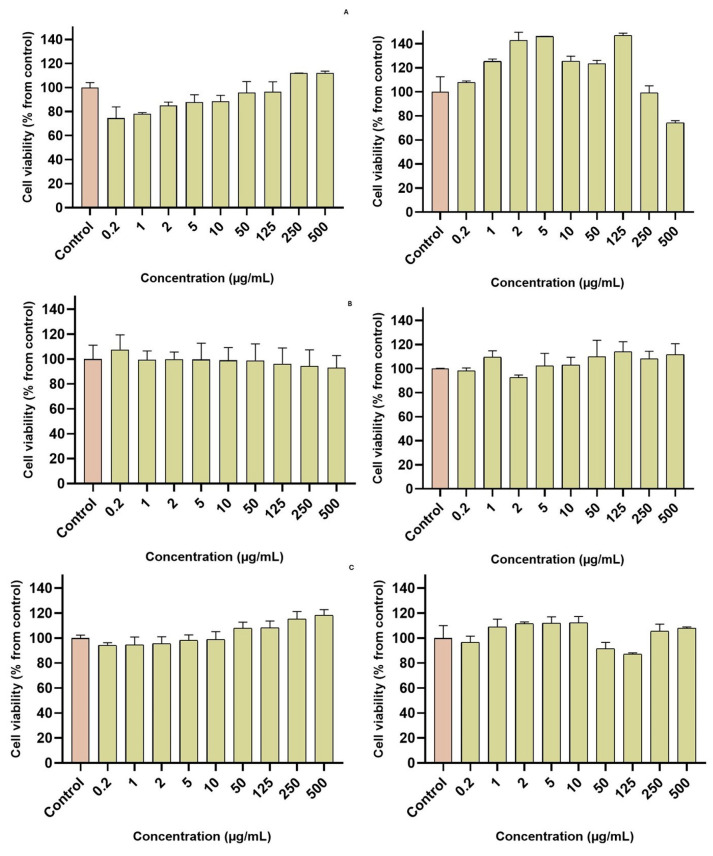
Cell viability of MRC-5 (**left**) and U-87 (**right**) cells exposed to SCG40 °C extract (**A**) and SCG40 °C-nZVI (**B**), and C-nZVI (**C**).

The cell viability of MRC-5 and U-87 cell lines exposed to SCG40 °C extract and nZVI was assessed. The results are presented in Figure 8. Cell lines were exposed to concentrations of extract and nZVI between 0.2 and 500 µg/mL. For the MRC-5 cell lines exposed to SCG40 °C extract, cell viability was slightly decreased in lower concentrations, but at higher concentrations was above 100%. For SCG40 °C-nZVI and C-nZVI, the cell viability remained above 90% in all tested concentrations. For the U87 cell line, the SCG40 °C extract influenced cell proliferation at 500 µg/mL, with cell viability dropping to 74%. The SCG40 °C-nZVI and C-nZVI did not show significant impact on cell viability, with cell values above 80% for all tested concentrations.

Nadagouda et al. [64] synthesized nZVI from tea polyphenols and assessed their cytotoxicity towards HaCaT cells, at concentrations between 5 and 100 µg/mL. The samples were tested after 24 and 48 h exposure time, showing no decrease in cell proliferation. Yu et al. [65] developed dopamine modified nZVI for photothermal and photodynamic breast cancer therapy and tested them on Human BC MCF-7 cells and normal BEAS-2B cells at concentrations between 5 and 100 µg/mL. The authors reported the modified nZVI’s tumoricidal ability, while retaining good biocompatibility to normal cells. Likewise, Chen et al. [66] demonstrated that green nZVIs produced from *Stachys lavandulifolia* Vahl. were cytotoxic against HSkMC (fibroblast cancer cell line) but not against normal (HUVEC) cell line.

### 3.12. Remediation Assays

**Figure 9:**
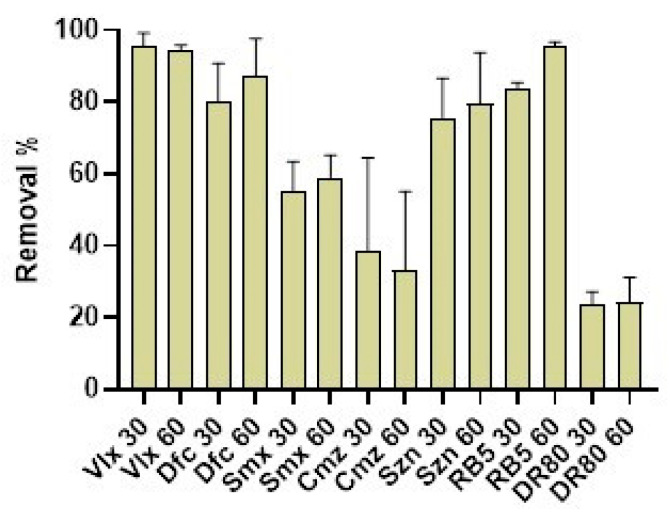
Removal % of contaminants by the presence of SCG40 °C-nZVI.

The remediation potential of SCG40 °C-nZVI was assessed, with seven different contaminants being tested. The results are presented in Figure 9. The best removal rates were obtained for Vlx, where up to 95% removal was obtained after only 30 min. For RB5, the removal increased from 83 to 95% between 30 and 60 min. For Dfc, removal rates above 80% were obtained for both times of contact. Szn and Smx displayed lower removal rates, above 75 and 50%, respectively. SCG40 °C-nZVI did not show good removal potential for Cmz and DR80, with removal rates below 40 and 30%, respectively.

Masud et al. [67] synthesized nZVI hybrids with graphene oxide and utilized them for the removal of several contaminants. At a concentration of 200 ppb, the nanohybrids displayed a removal rate of over 95% for Dfc, above 80% for Vlx and Cmz, and 74% for Smx, after 30 min. The application of advanced oxidation processes, combined with the nanohybrids, increased the removal rates to above 90% for all contaminants. Guo et al. [68] attempted to remove Szn (20 µM) using microscale ZVI produced by ball milling. The authors also synthesized ZVI modified with sulfidate and persulfate (PDS). Without persulfate, bare ZVI and sulfidated ZVI did not remove any Szn, but when PDS was used, degradation of 39.9% and 87.7% were achieved for PDS-ZVI and sulfidated PDS-ZVI. Shanableh et al. [69] investigated Smx removal by nZVI. The authors investigated the removal at different pH levels, and different mass ratios, with both factors influencing Smx removal. At a mass ratio of 10:1 nZVI to Smx, acidic conditions resulted in better removals (83 to 91%) than neutral or alkaline conditions (29% and 6%, respectively).

These results show the potential of nZVI and its composites for the removal of several contaminants. It is of importance to note that pH, nZVI mass, contaminant concentration, and the type of composite play a pivotal role in the removal of specific contaminants.

**Figure 10:**
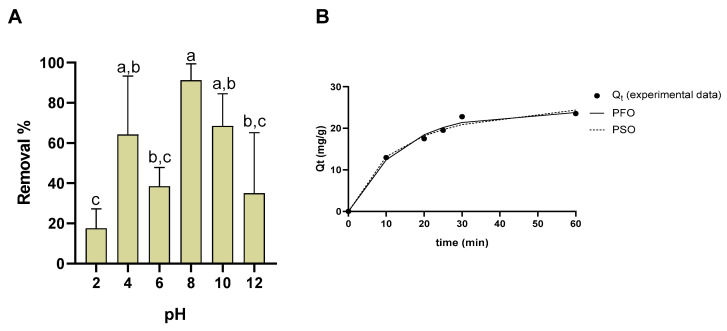
Removal % of Vlx by the presence of SCG40 °C-nZVI. (**A**)—Variation in removal % at different pH (different lowercase letters mean statistically significant differences at *p* > 0.05.); (**B**)—variation in removal % along time. PFO—pseudo-first order model; PSO—pseudo-second order model.

A study of pH was conducted, varying pH from 2 to 12. Results are presented in Figure 10A. At pH 8, the best adsorption rate was obtained. A great variation in the adsorption rate was observed, varying from 95% at pH 8, to below 40 and 20% at pH 12 and 2, respectively.

The kinetic study is depicted in Figure 10B, along with the adjusted model curves. For the pseudo-first order model, q_e_ = 2.41 × 10^1^ mg/g and k_1_ = 7.25 × 10^−2^ min^−1^ (SSE = 3.71 × 10^0^). For the pseudo-second order model, the best fit (SSE = 5.12 × 10^0^) was found to be at q_e_ = 2.93 × 10^1^ mg/g and k_2_ = 2.82 × 10^−3^ g.mg^−1^.min^−1^.

## 4. Conclusions

This study was conducted with SCG extracts for a sustainable green method of nZVI synthesis. The application of the extracts proves that nZVIs were successfully produced. Furthermore, a broad-scale characterization of the extract and the synthesized nZVIs was performed and included biochemical, physical and chemical analyses. The chemical characterization revealed that FTIR analysis confirmed that active components from SCGs were present in the nZVI. SEM-EDS displayed the spherical shape and size below 100 nm of the nZVI. A considerable difference in iron content between the SCG-nZVI and C-nZVI was observed (16% vs. 80%) due to the absence of organic matter in the latter. nZVI displayed magnetic properties, and no significant impact on cell viability. The use of SCGs to synthesize nZVI is a cost and environmentally friendly approach, that can be used to give use to otherwise wasted SCGs and diminish the use of toxic solvents in nZVI production. SCG40 °C-nZVI displayed very good removal efficiencies for Vlx, Dfc, and Rb5, which highlights its potential as a remediation agent. Further studies were performed for the removal of Vlx. More research is required on these NPs to assess their stability, biodegradability, potential environmental impact, and expand the knowledge on their remediation abilities.

## Figures and Tables

**Table 1:** Quantification of identified compounds in SCG extracts (mg/g extract dw).

	Compound	RT (min)	SCG40 °C	SCG60 °C	*p*-Value
**1**	**Trigonelline**	5.40	11.59 ± 0.20	10.60 ± 0.07	0.0012
**2**	**3-O-CQA**	10.85	7.49 ± 0.05	6.15 ± 0.10	<0.0001
**3**	**4-O-CQA**	15.60	11.90 ± 0.12	9.93 ± 0.04	<0.0001
**4**	**5-O-CQA**	17.10	11.29 ± 0.30	9.61 ± 0.30	0.0009
**5**	**4-O-FQA**	21.65	12.58 ± 0.20	10.99 ± 0.27	0.0013
**6**	**Caffeine**	24.60	47.50 ± 0.76	41.68 ± 0.18	0.0002
**7**	**5-O-FQA**	24.76	12.97 ± 0.39	11.73 ± 0.26	0.0103
**8**	**3,4-di-O-CQA**	33.88	0.61 ± 0.02	0.49 ± 0.01	0.0005
**9**	**3,5-di-O-CQA**	37.05	0.32 ± 0.03	0.28 ± 0.02	n.s.
**10**	**4,5-di-O-CQA**	44.14	2.77 ± 0.04	2.53 ± 0.06	0.0041
		**Total**	**119.02**	**103.99**	

**Table 2:** Phenolic content and antioxidant activity of the SCG extracts.

Sample	TPC (mg GAE/g dw)	DPPH^•^ (mg TE/g dw)	ABTS^•+^ (mg TE/g dw)	FRAP (mg AAE/g dw)
**SCG40 °C**	134.64 ± 14.73 ^a^	142.09 ± 40.59 ^a^	219.44 ± 26.61 ^a^	87.79 ± 6.65 ^a^
**SCG60 °C**	104.30 ± 14.56 ^b^	120.16 ± 23.80 ^b^	193.32 ± 59.10 ^a^	80.02 ± 15.55 ^a^

Different superscript lowercase letters (^a,b^) in the same column means statistically significant differences at *p* < 0.05.

**Table 3:** DLS measurements of SCG nZVI.

Sample	Size (nm)	PDI	ZP (mV)
**SCG40 °C-nZVI w**	565.60 ± 80.84 ^b^	0.56 ± 0.08 ^a^	−19.57 ± 0.95 ^a^
**SCG40 °C-nZVI w T**	14.64 ± 0.76 ^c^	0.24 ± 0.07 ^b,c^	−5.99 ± 1.71 ^b^
**SCG-40 °C-nZVI met**	514.30 ± 135.39 ^b^	0.43 ± 0.08 ^a,b^	−6.72 ± 2.76 ^b^
**SCG-40 °C-nZVI met T**	2112.33 ± 483.02 ^a^	0.52 ± 0.14 ^a^	−4.23 ± 0.19 ^b^
**SCG-60 °C-nZVI w T**	22.68 ± 6.79 ^c^	0.24 ± 0.08 ^c^	−6.97 ± 1.15 ^b^
**C-nZVI w**	868.16 ± 142.12 ^b^	0.57 ± 0.02 ^a^	−18.36 ± 1.34 ^a^
**C-nZVI w T**	466.86 ± 24.52 ^b,c^	0.48 ± 0.12 ^a^	−1.13 ± 0.24 ^c^

Abbreviations: met—methanol; T—Tween^®^20; w—water. Different superscript lowercase letters (^a,b,c^) in the same column means statistically significant differences at *p* < 0.05.

## Data Availability

The original contributions presented in this study are included in the article/Supplementary Material. Further inquiries can be directed to the corresponding author.

## Supplementary Materials

The following supporting information can be downloaded at https://www.mdpi.com/article/10.3390/nano15231788/s1, Figure S1: UV-Vis analysis of SCG40 °C-nZVI (top), and SCG40 °C-nZVI with Tween^®^ 20 added before (B4) and after (Af) synthesis and kept at 4 °C and at 20 °C.; Figure S2: FTIR analysis of SCG40 °C-nZVI (top), and SCG40 °C-nZVI with Tween^®^ 20 added before (B4) and after (Af) synthesis and kept at 4 °C and at 20 °C.; Figure S3: Sedimentation of nZVI. A—nZVI after synthesis; B—nZVIs after 1 day; C—nZVIs after 1 week.; Table S1: Calibration curves of compounds found in SCG extracts.
