# Supplementary material for: Upcycling Coffee Waste into Sustainable Nano Zerovalent Iron for Environmental Contaminant Remediation: Characterization, Applicability and Cytotoxicity
Source: Nanomaterials (Basel). 2025 Nov 27;15(23):1788. doi: 10.3390/nano15231788 (PMC12693293; doi:10.3390/nano15231788)
Supplement: Supplementary file 1 [file nanomaterials-15-01788-s001.zip › nanomaterials-4006811-supplementary.pdf]

Supplementary Materials

Upcycling Coffee Waste into Sustainable Nano Zerovalent Iron for Environmental Contaminant Remediation: Characterization, Applicability and Cytotoxicity

Filipe Fernandes [1,2], Maria Freitas [1], Cláudia Pinho [3], Ana Isabel Oliveira [3], Cristina Delerue-Matos [1] and Clara Grosso [1,*]

[1] REQUIMTE/LAQV, ISEP, Polytechnic of Porto, Rua Doutor António Bernardino de Almeida, 431, 4249-015 Porto, Portugal
[2] Faculty of Sciences, University of Porto, Rua do Campo Alegre, s/n, 4169-007 Porto, Portugal
[3] REQUIMTE/LAQV, School of Health, Rua Doutor António Bernardino de Almeida, 400, 4200-072 Porto, Portugal
* Correspondence: claragrosso@graq.isep.ipp.pt

Table S1. Calibration curves of compounds found in SCG extracts.

	Compound	Equation	R^2	LOD (mg/mL)	LOQ (mg/mL)
1	**Trigonelline**	$y=3.29\times10^7x-2.48\times10^4$	0.9999	1.06×10^{-4}	3.21×10^{-4}
2	**3-*O*-CQA**	$y=7.20\times10^7x-7.30\times10^4$	0.9995	1.48×10^{-5}	4.48×10^{-5}
3	**4-*O*-CQA**	$y=6.18\times10^7x-3.99\times10^4$	0.9998	1.77×10^{-5}	5.35×10^{-5}
4	**5-*O*-CQA**	$y=7.19\times10^7x-2.76\times10^4$	1.000	7.19×10^{-7}	6.45×10^{-5}
5	**4-*O*-FQA**	$y=2.26\times10^7x-3.31\times10^4$	0.9995	5.36×10^{-5}	1.62×10^{-4}
6	**Caffeine**	$y=6.18\times10^7x-2.07\times10^4$	0.9999	5.20×10^{-6}	1.58×10^{-5}
7	**5-*O*-FQA**	$y=2.31\times10^7x-1.30\times10^4$	0.9997	1.35×10^{-5}	4.08×10^{-5}
9	**3,5-di-*O*-CQA**	$y=9.28\times10^7x+1.23\times10^5$	0.9950	3.11×10^{-5}	9.43×10^{-5}
10	**4,5-di-*O*-CQA**	$y=5.12\times10^7x+7.72\times10^4$	0.9939	2.98×10^{-5}	9.04×10^{-5}

The limit of detection (LOD) and limit of quantification (LOQ) were determined using the residual standard deviation (σ) of the regression curve and the slope (S) of the calibration curve, applying the following equations: LOD = 3.3σ/S and LOQ = 10σ/S.

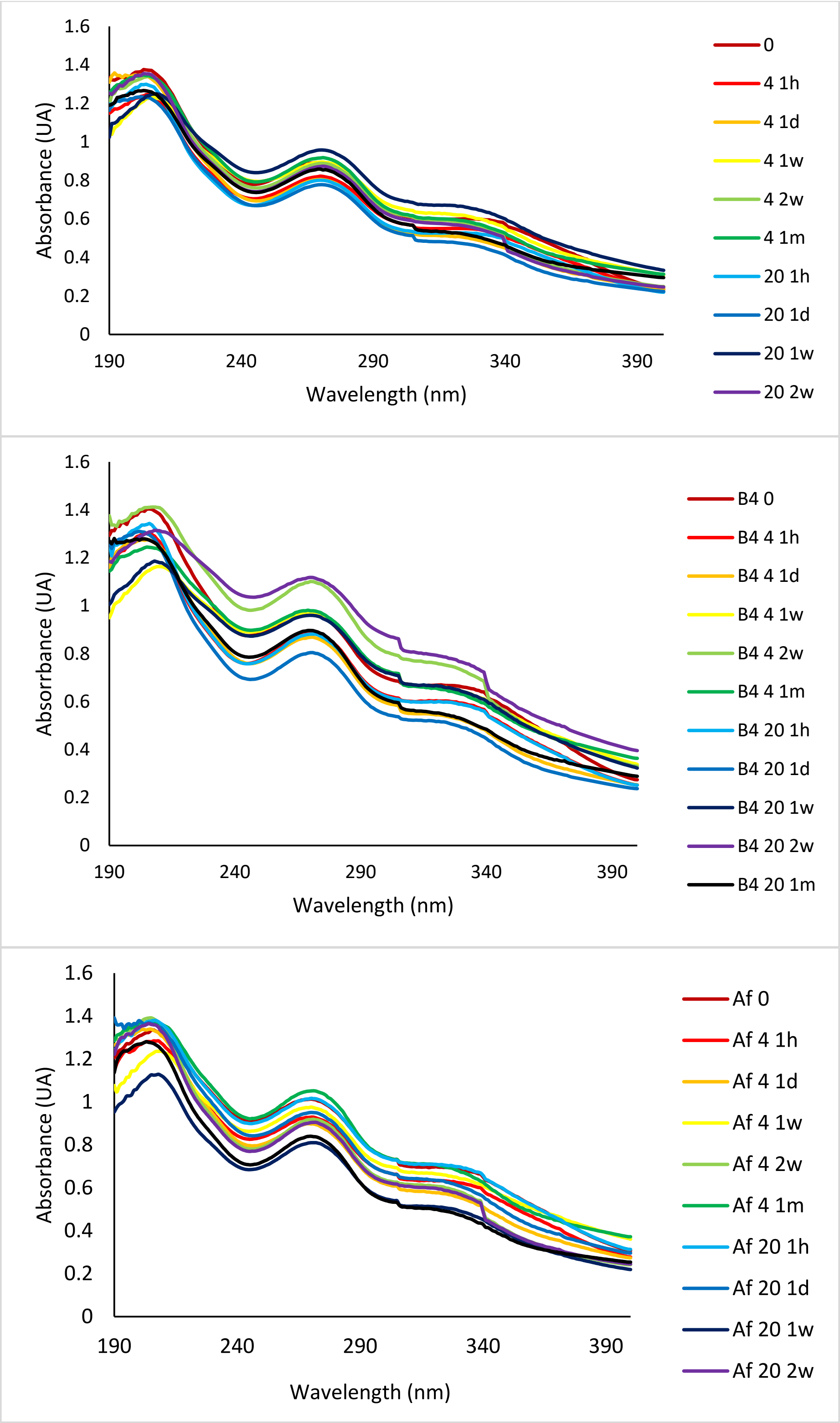

Figure S1. UV-Vis analysis of SCG40°C-nZVI (top), and SCG40°C-nZVI with Tween® 20 added before (B4) and after (Af) synthesis and kept at 4 °C and at 20 °C.

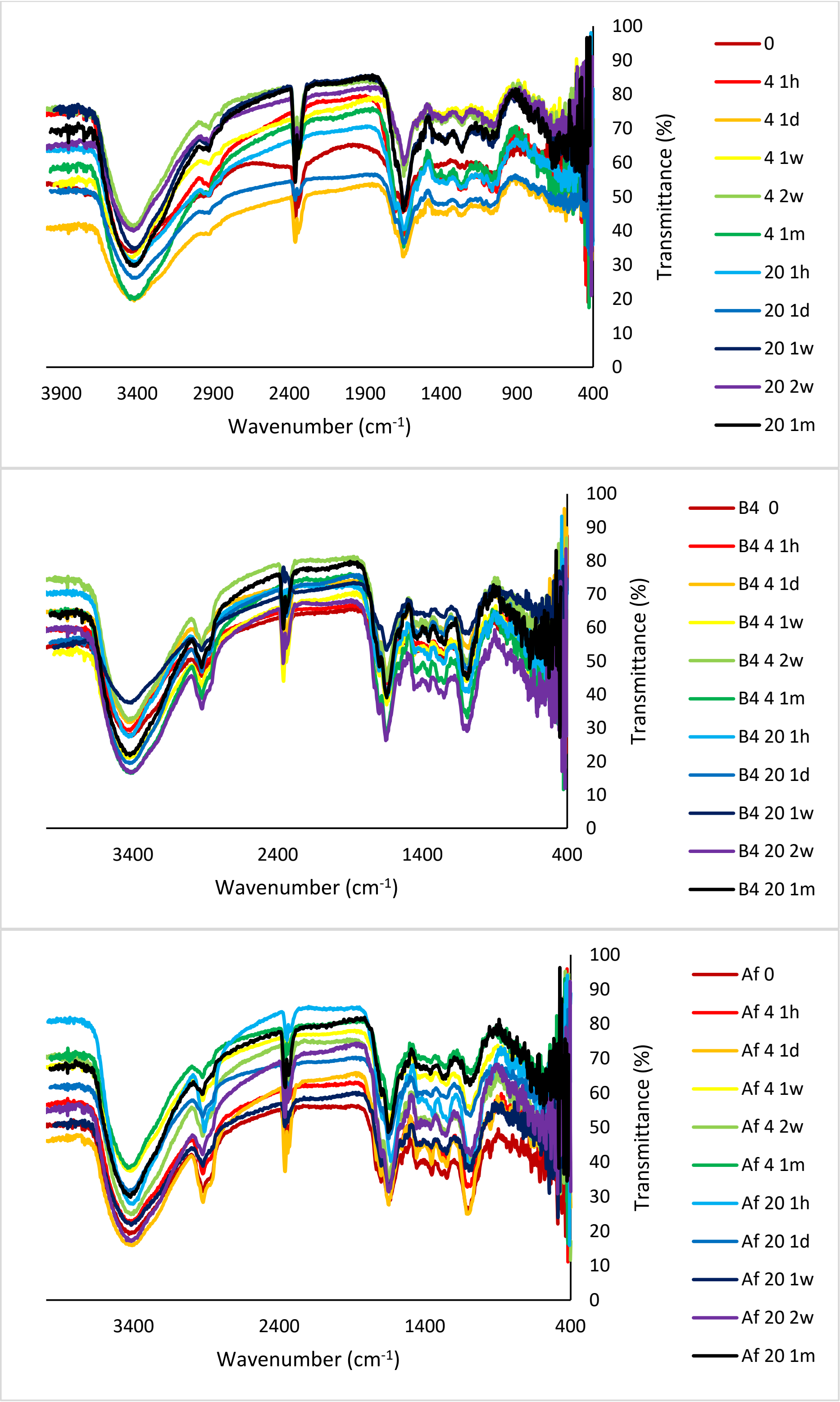

Figure S2. FTIR analysis of SCG40°C-nZVI (top), and SCG40°C-nZVI with Tween® 20 added before (B4) and after (Af) synthesis and kept at 4 °C and at 20 °C.

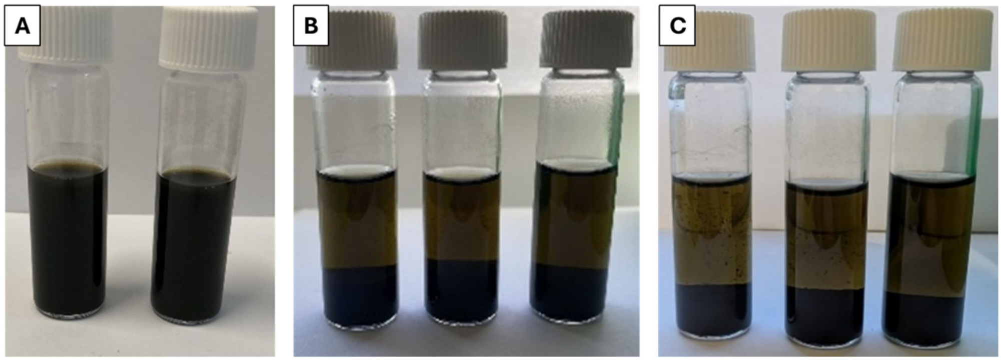

Figure S3. Sedimentation of nZVI. A - nZVI after synthesis; B - nZVIs after 1 day; C - nZVIs after 1week.
